# Association of co-occurrence of gastrointestinal, sleep, and affective symptoms with *Helicobacter pylori* infection: a monocentric cross-sectional study in China

**DOI:** 10.3389/fendo.2025.1675866

**Published:** 2025-11-07

**Authors:** ZhiHai Zhang, HongFei Ke, XiaoYan ShangGuan, NengJiang Zhao, Xin Hu, Bo Li, MiaoNa Cai, MeiQi Chen, JiaHao Liu, JieLong Wu, ShuYu Yang

**Affiliations:** 1 Center of Integrated Chinese and Western Medicine, The First Affiliated Hospital of Xiamen University, School of Medicine, Xiamen University, Xiamen, Fujian, China; 2 The First Affiliated Hospital of Xiamen University, School of Medicine, Xiamen University, Xiamen, Fujian,, China; 3 Guangdong Metabolic Disease Research Center of Integrated Chinese and Western Medicine, Guangdong Pharmaceutical University, Guangzhou, China

**Keywords:** *Helicobacter pylori*, extra-gastric manifestations, sleep disturbances, affectivedisorders, triadic symptom cluster

## Abstract

**Background:**

*Helicobacter pylori* (*H. pylori*) is a globally prevalent gastric bacterium implicated in gastrointestinal disease. Emerging evidence suggests it may also contribute to extra-gastric manifestations; however, its relationship with concurrent gastrointestinal, psychological, and sleep disturbances remains underexplored. This study investigated whether *H. pylori* infection is associated with a triad of gastrointestinal discomfort, sleep disturbance, and affective disorders.

**Methods:**

This single-center cross-sectional study enrolled 969 adults at a *H. pylori* detection clinic (First Affiliated Hospital of Xiamen University) between June 2023 and June 2024. *H. pylori* infection status and severity were assessed using a 13C-urea breath test. Gastrointestinal symptoms, sleep disturbances, and affective disorders were evaluated with the Gastrointestinal Symptom Rating Scale (GSRS), Athens Insomnia Scale (AIS), and 4-item Patient Health Questionnaire (PHQ-4). Latent class analysis (LCA) was performed to identify distinct symptom phenotypes. Associations between infection severity and symptom clusters were examined using logistic regression adjusted for age, sex, and body mass index (BMI).

**Results:**

In terms of associated symptoms, 35.40% of participants reported gastrointestinal symptoms, 72.65% had sleep disturbances, and 61.09% experienced affective disorders. The prevalence of gastrointestinal symptoms, sleep disturbances, and affective disorders increased significantly with greater infection severity. LCA identified a “Positive Triad” phenotype (60.7% of the cohort), characterized by concurrent gastrointestinal, sleep, and emotional symptoms. Higher infection severity was independently associated with this combined symptom cluster after adjusting for age, sex, and BMI. The effect of *H. pylori* infection on risk of positive triad was significant among different gender, age group, and BMI group.

**Conclusion:**

*H. pylori* infection was associated with concurrent gastrointestinal discomfort, sleep disturbance, and affective disorders. These findings suggest a unified symptom cluster attributable to *H. pylori* infection, extending its clinical impact beyond the gastric tract. Recognizing this multidimensional presentation could inform more comprehensive diagnostic and therapeutic strategies for affected patients.

## Introduction

*Helicobacter pylori* (*H. pylori*) infection remains one of the most prevalent gastrointestinal infections worldwide, affecting over half of the global population, with particularly high burden in developing countries (1). Its established role in chronic gastritis, peptic ulcer disease, mucosa-associated lymphoid tissue (MALT) lymphoma, and gastric carcinoma is well documented through histopathological and molecular evidence (2). Beyond its well-characterized gastric effects, a growing body of research has suggested that *H. pylori* infection may exert systemic influences, extending its clinical relevance beyond the gastrointestinal tract.

In recent years, attention has turned toward the potential extra-gastric manifestations of *H. pylori*, particularly its associations with functional gastrointestinal disorders, sleep disturbances, and affective symptoms such as anxiety and depression. Epidemiological data indicate that *H. pylori*-positive individuals report higher rates of dyspepsia, altered gastric motility, insomnia, and affective disorders compared to uninfected counterparts (3–5). These findings suggest a broader clinical impact of *H. pylori* that spans digestive, neurological, and psychological domains.

Mechanistically, chronic *H. pylori* colonization can trigger persistent mucosal inflammation, elevate systemic pro-inflammatory cytokines such as IL-6 and TNF-α, and compromise gastrointestinal barrier integrity, collectively altering gut-brain axis signaling and neurotransmitter regulation (6–9). These alterations may influence sleep regulation, mood stability, and visceral sensitivity via neuroimmune and neuroendocrine pathways (10–13). However, although these domains—gastrointestinal symptoms, sleep disturbance, and psychological distress—have been individually linked to *H. pylori*, relatively few studies have examined whether they may co-occur as a unified symptom constellation.

In clinical practice, it is not uncommon to encounter patients simultaneously experiencing dyspeptic complaints, insomnia, and emotional instability. Drawing from this observation, the present study aimed to investigate the co-occurrence of these three symptom domains and examine their relationship with *H. pylori* infection status. We hypothesized that *H. pylori* infection is significantly associated with a functional symptom triad comprising gastrointestinal discomfort, sleep disturbance, and affective disorders. Confirmation of such an association would have important implications for both the understanding and management of *H. pylori*-related morbidity.

## Materials and methods

### Study design and participants

This single-center cross-sectional study enrolled participants who visited the *H. pylori* detection clinic at the First Affiliated Hospital of Xiamen University between June 2023 and June 2024. The study was approved by the Medical Ethics Committee of the First Affiliated Hospital of Xiamen University(Approval number: [2022] Scientific Research and Regulations (066)), and all participants provided informed consent. Exclusion criteria were as follows: (1) pregnant individuals; (2) participants with previous psychiatric diagnoses; (3) participants who had used antibiotics or proton pump inhibitors (PPIs) within 4 weeks prior to enrollment; and (4) participants unable to complete the assessment scales. A total of 969 participants were ultimately included in the study.

### Sample size considerations

In current study, the sample size was calculated based on an estimated *H. pylori* infection rate (p) of 0.8, with an allowable error (d) of 0.04 and a two-sided significance level (ɑ) of 0.05. The sample size calculation formula is as follows: $n\;=\;\frac{z_{\alpha }^{2}\times P\times \left(1-P\right)}{d^{2}}$. Under these parameters, a minimum sample size of 969 participants ensures the required accuracy and achieves a statistical power of 85.99% for estimating the infection rate using a two-sided exact test.

### Determination of *H. pylori* infection

*H. pylori* infection was detected by carbon-13 urea breath test (^13^C-UBT)(Shenzhen Zhonghe Headway Bio-Sci & Tech Co., Ltd), which is widely considered as one of the most accurate, non-invasive tests for *H. pylori* infection detection (14–18). Participants were instructed to ingest a 13C-labeled urea capsule, with exhaled breath samples collected at baseline and 30 minutes post-ingestion for *H. pylori* detection. All participants underwent standardized testing using the Shenzhen Zhonghe Headway Bio-Sci & Tech Co., Ltd. Breath Test System (Breath Tester HCBT-01 model) Based on the results of the ^13^C-UBT, and the interpretation guidelines and user manual provided by the manufacturer, a score of ≤4 indicates a negative result for *H. pylori* infection, while scores of 4–10 represent mild infection, 10–20 indicate moderate infection, and >20 signify severe infection.

### Variables of interest

This study collected basic demographic characteristics of the participants, including gender, age, and BMI. In addition, we gathered information on gastrointestinal symptoms, sleep disturbances, and affective disorders. Gastrointestinal symptoms were assessed using the Gastrointestinal Symptom Rating Scale (GSRS) (**Supplementary Table 1**), which consists of 15 items with a total score ranging from 0 to 75. A score of ≥31 indicates that intervention is needed and is classified as having gastrointestinal symptoms (18). Sleep disturbances were evaluated using the Athens Insomnia Scale (AIS) (**Supplementary Table 2**), which comprises 8 items with a total score between 0 and 24; a score of ≥5 indicates that intervention is warranted and is classified as having sleep disturbances (19). Affective disorders were examined with the 4-item Patient Health Questionnaire (PHQ-4) (**Supplementary Table 3**), which includes both a depression scale (PHQ-2) and an anxiety scale (GAD-2), with a total score ranging from 0 to 6. A score of ≥3 suggests the need for intervention and is classified as having affective disorders (20).

### Statistical analysis

Descriptive statistical analyses were first conducted to summarize participants’ sociodemographic and disease characteristics according to their *H. pylori* infection status. Differences among the four *H. pylori* infection groups were assessed using ANOVA for continuous variables and the chi-square test for categorical variables. When overall differences were significant, *post hoc* pairwise comparisons were performed using the Bonferroni correction. Second, scatter plots were generated to illustrate the relationships between the ^13^C-UBT results and the GSRS, AIS, and PHQ-4 scores, followed by Spearman correlation analyses to assess the strength and significance of these associations. Correlation heat maps were subsequently created to explore interrelationships among demographic characteristics, ^13^C-UBT results, and GSRS, AIS, and PHQ-4 scores.

Third, latent class analysis (LCA) was performed using the poLCA package in R to identify the triad of symptoms (gastrointestinal symptoms, sleep disturbances, and affective disorders). The optimal number of latent classes was determined by model fit statistics (e.g., Akaike Information Criterion [AIC] and Bayesian Information Criterion [BIC]). Conditional item response probabilities for each symptom across the latent classes were then visualized, and the identified classes were incorporated into the final analysis dataset.

Fourth, multivariate logistic regression analyses were used to examine associations between *H. pylori* infection and the variables of interest, including gastrointestinal symptoms, sleep disturbances, affective disorders, and the triad of symptom latent classes. Models were adjusted for gender, age, and BMI as covariates to account for potential confounding factors that could influence the relationship between H. pylori infection and the outcomes. Odds ratios (ORs) and 95% confidence intervals (CIs) were calculated by coding *H. pylori* infection as a continuous variable (i.e., normal, mild, moderate, and severe infection as 1, 2, 3, and 4, respectively). Subgroup analyses were also conducted to investigate whether the relationship between *H. pylori* infection and the triad of symptoms varied by gender, age group, and BMI group.

All statistical tests were two-tailed, and a *p*-value< 0.05 was considered statistically significant. Data organization and analyses were carried out using R software (version 4.4.1, Posit Software, PBC, Boston, MA, USA).

## Results

### Characteristics of the study population

A total of 969 participants were included in the study, with an average age of 37.07 ± 12.55 years, of whom 71.10% were female. The mean BMI was 21.99 ± 3.77 kg/m², and the average value of ^13^C-UBT results was 23.86 ± 18.40. Based on the classification of ^13^C-UBT results, the number of participants with normal, mild, moderate, and severe *H. pylori* infections were 182, 40, 217, and 530, respectively. Female participants and individuals with lower BMI exhibited significantly higher levels of *H. pylori* infection severity (*p* < 0.05).

In terms of associated symptoms, 35.40% of participants reported gastrointestinal symptoms, 72.65% had sleep disturbances, and 61.09% experienced affective disorders. The prevalence of gastrointestinal symptoms, sleep disturbances, and affective disorders increased significantly with the severity of *H. pylori* infection, with notable differences in each specific item across the scales (*p* < 0.001). Symptom severity was significantly higher in patients with moderate and severe *H. pylori* infection than in those with normal findings or mild infection (*p* < 0.05). Detailed data can be found in **Table 1**.

**Table 1:** Individuals’ sociodemographic and disease characteristics.

Characteristic	Overall	*H. pylori* infection				*p*-value^2^
		Normal	Mild	Moderate	Severe	
	N = 969^1^	N = 182(18.8%)^1^	N = 40(4.1%)^1^	N = 217(22.4%)^1^	N = 530(54.7%)^1^	
¹³C UBT results	23.86 ± 18.40	0.78 ± 0.75^bcd^	6.99 ± 1.74^acd^	15.30 ± 2.53^abd^	36.56 ± 14.82^abc^	<0.001
Gender						<0.001
Male	280 (28.90%)	70 (38.46%)^d^	21 (52.50%)^d^	77 (35.48%)^d^	112 (21.13%)^abc^	
Female	689 (71.10%)	112 (61.54%)	19 (47.50%)	140 (64.52%)	418 (78.87%)	
Age (years)	37.07 ± 12.55	38.48 ± 14.21	38.48 ± 14.13	35.50 ± 12.12	37.12 ± 11.94	0.131
BMI (Kg/m^2)	21.99 ± 3.77	22.42 ± 3.89^d^	22.75 ± 4.41	22.63 ± 4.11^d^	21.53 ± 3.48^ac^	<0.001
Gastrointestinal disorders	343 (35.40%)	3 (1.65%)^cd^	1 (2.50%)^cd^	109 (50.23%)^ab^	230 (43.40%)^ab^	<0.001
GSRS scores	22.07 ± 13.02	6.55 ± 8.23^cd^	8.50 ± 8.13^cd^	25.90 ± 11.33^ab^	26.85 ± 10.19^ab^	<0.001
Abdominal pains	1.15 ± 1.13	0.64 ± 1.06^cd^	0.73 ± 1.11^cd^	1.24 ± 1.04^ab^	1.31 ± 1.14^ab^	<0.001
Heartburn	1.56 ± 1.42	0.34 ± 0.89^cd^	0.40 ± 1.01^cd^	1.86 ± 1.39^ab^	1.95 ± 1.32^ab^	<0.001
Acid regurgitation	1.81 ± 1.35	0.60 ± 1.11^cd^	0.65 ± 0.98^cd^	2.12 ± 1.25^ab^	2.18 ± 1.20^ab^	<0.001
Sucking sensations in the epigastrium	1.47 ± 1.31	0.37 ± 0.94^cd^	0.48 ± 0.99^cd^	1.69 ± 1.23^ab^	1.84 ± 1.23^ab^	<0.001
Nausea and vomiting	1.16 ± 1.15	0.21 ± 0.74^cd^	0.28 ± 0.68^cd^	1.40 ± 1.08^ab^	1.46 ± 1.12^ab^	<0.001
Borborygmus	1.26 ± 1.17	0.20 ± 0.62^cd^	0.28 ± 0.64^cd^	1.60 ± 1.16^ab^	1.55 ± 1.09^ab^	<0.001
Abdominal distension	1.83 ± 1.39	0.92 ± 1.29^cd^	1.00 ± 1.28^cd^	2.00 ± 1.28^ab^	2.13 ± 1.32^ab^	<0.001
Eructation	1.69 ± 1.36	0.64 ± 1.18^cd^	0.50 ± 0.85^cd^	1.99 ± 1.31^ab^	2.02 ± 1.24^ab^	<0.001
Increased flatus	1.72 ± 1.42	0.43 ± 1.03^cd^	0.78 ± 1.12^cd^	2.10 ± 1.31^ab^	2.07 ± 1.30^ab^	<0.001
Decreased passage of stools	1.35 ± 1.37	0.40 ± 1.04^cd^	0.45 ± 0.93^cd^	1.61 ± 1.34^ab^	1.63 ± 1.34^ab^	<0.001
Increased passage of stools	1.39 ± 1.24	0.38 ± 0.91^cd^	0.50 ± 0.91^cd^	1.62 ± 1.11^ab^	1.71 ± 1.20^ab^	<0.001
Loose stools	1.51 ± 1.29	0.64 ± 1.13^cd^	0.98 ± 1.48^cd^	1.73 ± 1.22^ab^	1.76 ± 1.21^ab^	<0.001
Hard stools	1.30 ± 1.24	0.24 ± 0.83^cd^	0.28 ± 0.64^cd^	1.57 ± 1.21^ab^	1.64 ± 1.15^ab^	<0.001
Urgent need for defecation	1.50 ± 1.30	0.20 ± 0.62^cd^	0.70 ± 1.60^cd^	1.78 ± 1.11^ab^	1.89 ± 1.21^ab^	<0.001
Feeling of incomplete evacuation	1.45 ± 1.25	0.32 ± 0.83^cd^	0.53 ± 1.13^cd^	1.83 ± 1.16^ab^	1.75 ± 1.14^ab^	<0.001
Sleep Disturbance	704 (72.65%)	45 (24.73%)^cd^	13 (32.50%)^cd^	183 (84.33%)^ab^	463 (87.36%)^ab^	<0.001
AIS scores	6.03 ± 3.52	3.06 ± 3.38^cd^	3.50 ± 3.11^cd^	6.65 ± 3.13^ab^	6.99 ± 3.09^ab^	<0.001
Sleep induction	0.98 ± 0.84	0.55 ± 0.81^cd^	0.68 ± 0.83^cd^	1.01 ± 0.84^ab^	1.14 ± 0.80^ab^	<0.001
Awakenings during the night	0.72 ± 0.75	0.36 ± 0.59^cd^	0.30 ± 0.52^cd^	0.71 ± 0.73^ab^	0.87 ± 0.76^ab^	<0.001
Final awakening earlier than desired	0.63 ± 0.72	0.17 ± 0.47^cd^	0.20 ± 0.41^cd^	0.81 ± 0.70^ab^	0.74 ± 0.75^ab^	<0.001
Total sleep duration	0.79 ± 0.74	0.48 ± 0.68^cd^	0.55 ± 0.64^cd^	0.89 ± 0.74^ab^	0.88 ± 0.73^ab^	<0.001
Overall quality of sleep	0.80 ± 0.71	0.54 ± 0.67^cd^	0.55 ± 0.64^cd^	0.83 ± 0.71^ab^	0.89 ± 0.71^ab^	<0.001
Sense of well-being during the day	0.60 ± 0.69	0.13 ± 0.39^cd^	0.20 ± 0.41^cd^	0.71 ± 0.72^ab^	0.74 ± 0.70^ab^	<0.001
Functioning (physical and mental) during the day	0.71 ± 0.61	0.44 ± 0.56^cd^	0.38 ± 0.59^cd^	0.79 ± 0.59^ab^	0.80 ± 0.60^ab^	<0.001
Sleepiness during the day	0.81 ± 0.61	0.38 ± 0.60^bcd^	0.65 ± 0.66^ad^	0.91 ± 0.54^a^	0.93 ± 0.57^ab^	<0.001
Affective Disorders	592 (61.09%)	14 (7.69%)^cd^	8 (20.00%)^cd^	165 (76.04%)^ab^	405 (76.42%)^ab^	<0.001
PHQ2+GAD2 scores	2.89 ± 1.89	1.05 ± 1.36^cd^	1.23 ± 1.40^cd^	3.39 ± 1.84^ab^	3.44 ± 1.63^ab^	<0.001
Anhedonia	0.54 ± 0.71	0.10 ± 0.39^cd^	0.28 ± 0.60^cd^	0.63 ± 0.78^ab^	0.68 ± 0.72^ab^	<0.001
Depressed mood	0.70 ± 0.69	0.08 ± 0.36^cd^	0.15 ± 0.36^cd^	0.87 ± 0.70^ab^	0.89 ± 0.65^ab^	<0.001
Feeling nervous, anxious, or on edge	0.95 ± 0.74	0.79 ± 0.85^cd^	0.55 ± 0.78^cd^	1.06 ± 0.71^ab^	0.98 ± 0.68^ab^	<0.001
Not being able to stop or control worrying	0.69 ± 0.70	0.08 ± 0.34^cd^	0.25 ± 0.59^cd^	0.84 ± 0.69^ab^	0.88 ± 0.66^ab^	<0.001

*^1^*Mean ± SD; n (%).

*^2^*One-way analysis of means (not assuming equal variances); Pearson’s Chi-squared test; Fisher’s exact test.

Significant differences (*p* < 0.05, Bonferroni *post hoc* test): a vs. Normal; b vs. Mild; c vs. Moderate; d vs. Severe.

### Associations between ^13^C-UBT results and other variables

**Figure 1:**
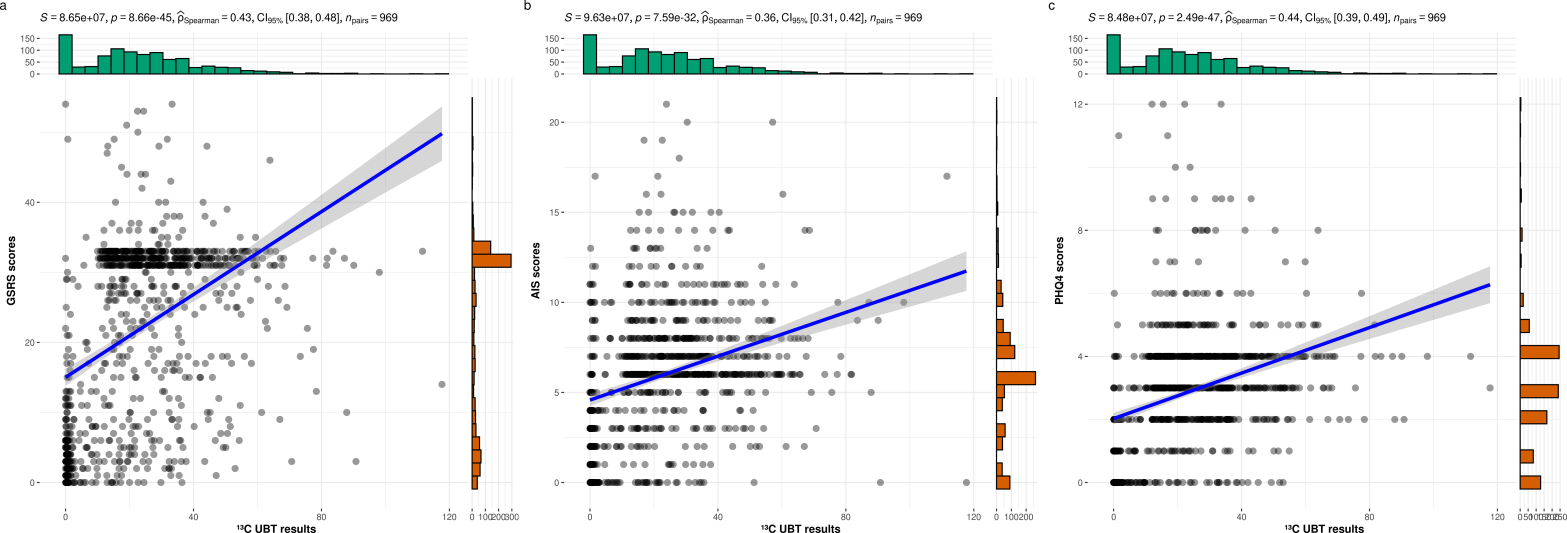
The relationships between the ^13^C-UBT results and the GSRS, AIS, and PHQ-4 scores. **(a)**^13^C-UBT results and GSRS score (*r* = 0.43, *p<* 0.001); **(b)**^13^C-UBT results and AIS score (*r* = 0.36, *p<* 0.001); **(c)**^13^C-UBT results and PHQ4 score (*r* = 0.44, *p<* 0.001).

The scatterplots in **Figure 1** demonstrate that as ^13^C-UBT results increase, participants exhibit significantly elevated GSRS, AIS, and PHQ-4 scores, with Spearman correlation coefficients of 0.43, 0.36, and 0.44, respectively. These findings suggest a significant association between *H. pylori* infection and gastrointestinal symptoms, sleep disturbances, and affective disorders.

**Figure 2:**
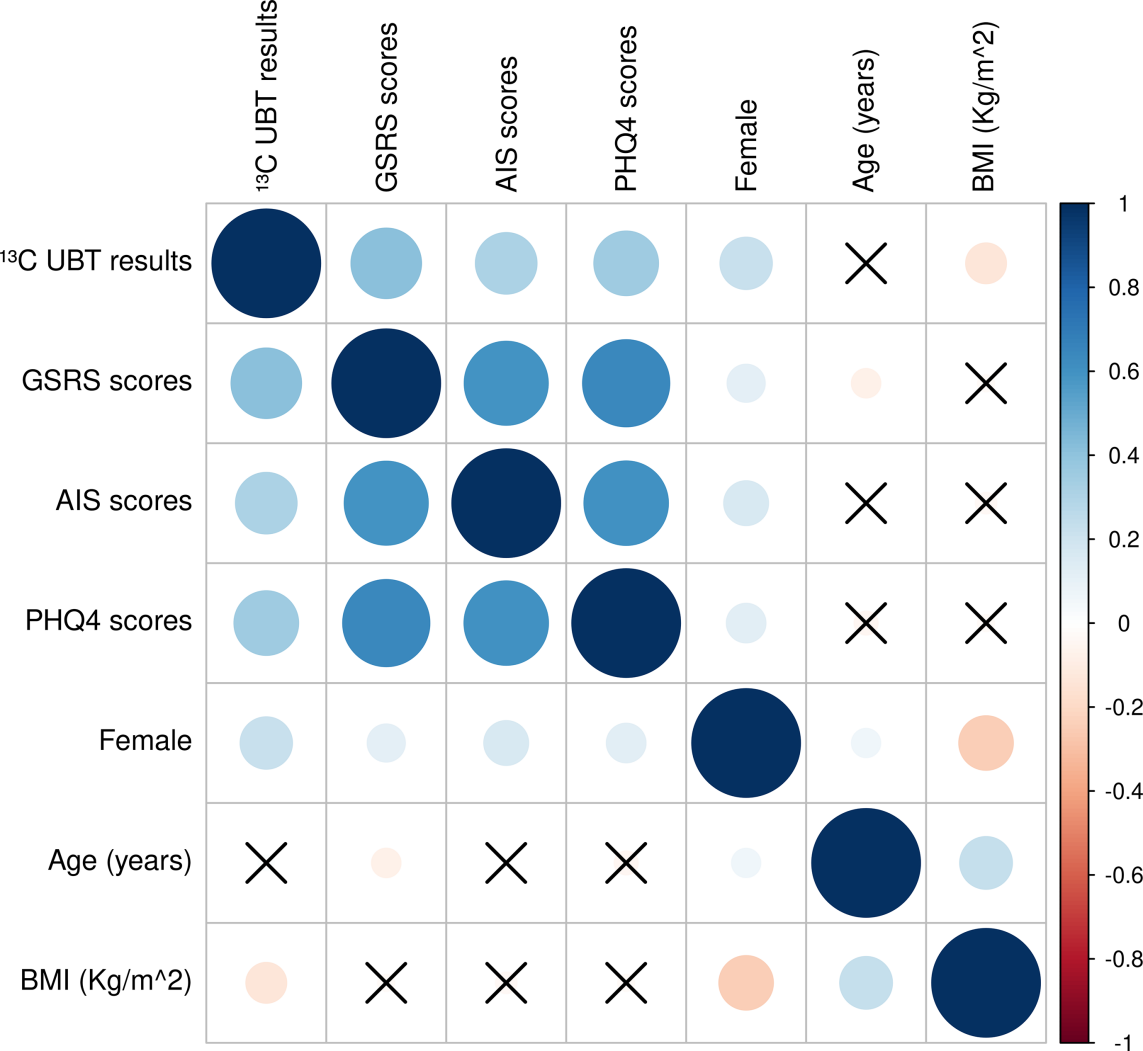
Correlation heat maps of demographics, ^13^C-UBT, GSRS, AIS, and PHQ-4 scores. Circles represent significant correlations and crosses represent insignificant correlations; blue circles are positive correlations and red circles are negative correlations; correlation coefficients range from -1 to 1, with larger circles representing larger correlation coefficients.

**Figure 2** further illustrates significant positive correlations between all pairs of variables (^13^C-UBT results, GSRS, AIS, and PHQ-4 scores), with female gender also showing significant associations with all these factors. These results highlight the importance of examining the triad of gastrointestinal symptoms, sleep disturbances, and affective disorders as an interconnected phenomenon.

### Latent classes based on the triad of symptoms

**Figure 3:**
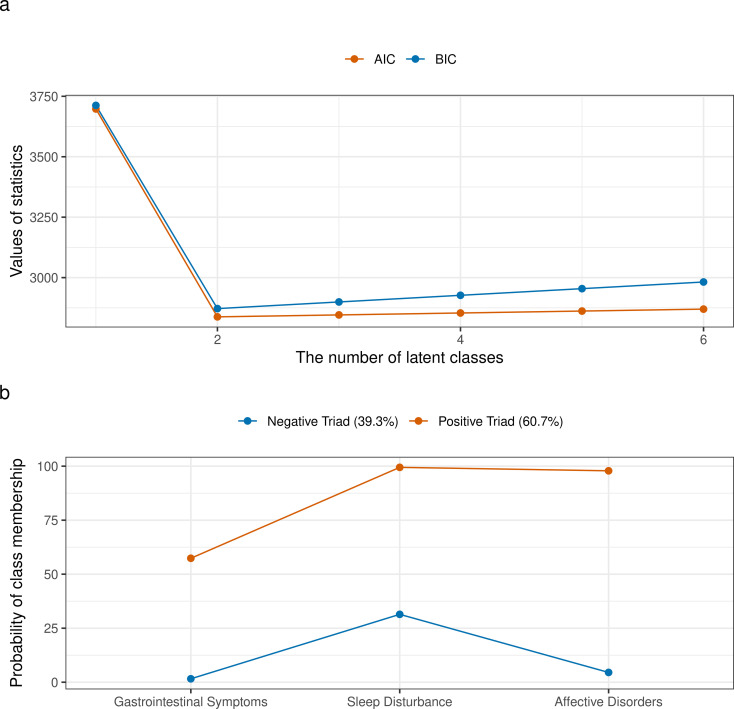
Identification of latent classes based on the triad of symptoms. **(a)** AIC and BIC values for models with varying numbers of latent classes, used to determine the optimal number of latent classes; **(b)** Conditional item response probabilities for each symptom across the identified latent classes.

The identification of latent classes based on the triad of symptoms is illustrated in **Figure 3**. When the number of latent classes was set to two, the model achieved the best fit, as evidenced by the lowest AIC and BIC values (**Figure 3a**). In this model, Latent Class 1 (referred to as the “Negative Triad”) exhibited low predicted probabilities for all three symptoms, whereas Latent Class 2 (referred to as the “Positive Triad”) demonstrated high predicted probabilities for all three symptoms (**Figure 3b**). A total of 39.3% of participants were classified as belonging to the “Negative Triad,” while 60.7% were classified as belonging to the “Positive Triad”.

### The association between *H. pylori* infection and different symptoms

**Figure 4:**
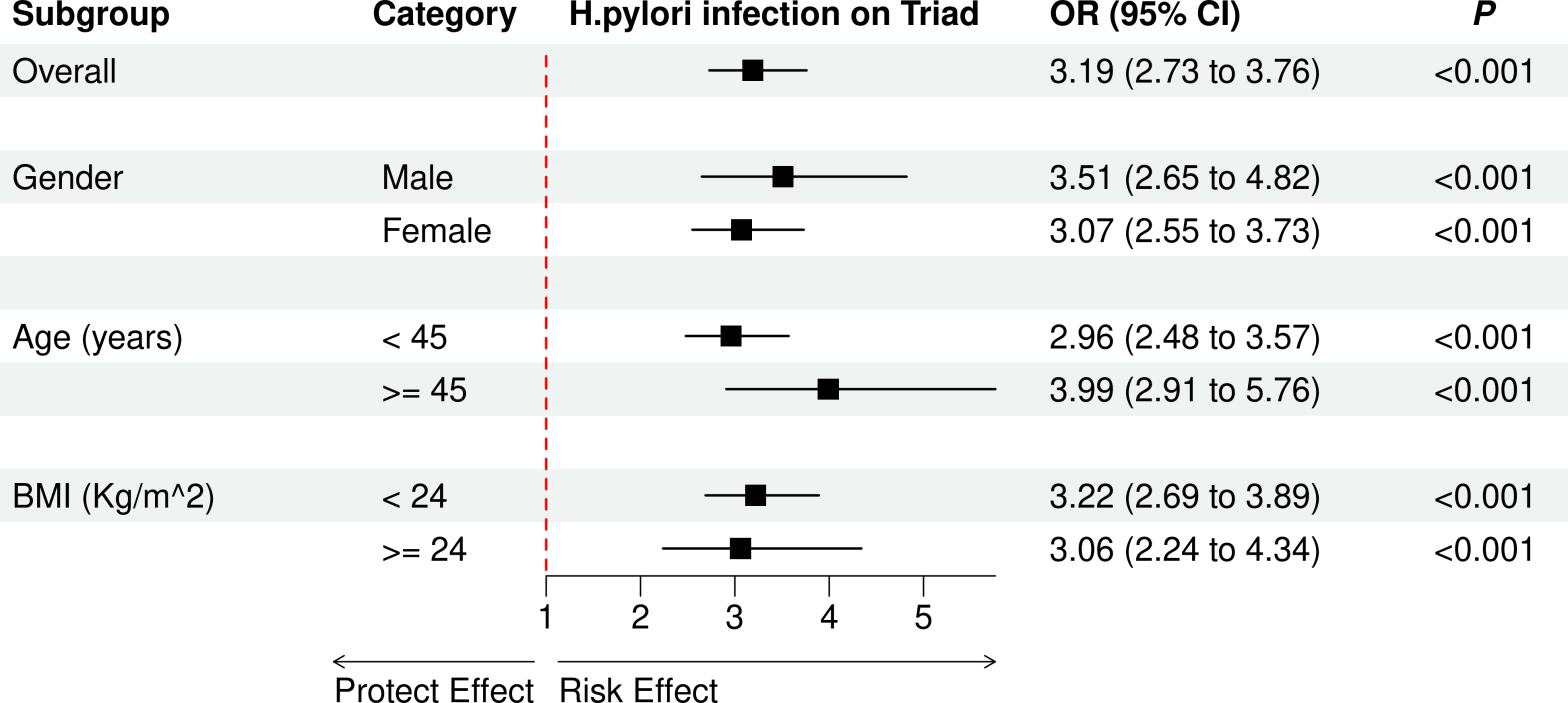
The effect of *H. pylori* infection on risk of positive triad in different subgroups. OR, odds ratio; CI, confidence interval.

**Table 2** demonstrates a significant association between *H. pylori* infection and different symptoms, including gastrointestinal symptoms, sleep disturbances, affective disorders, and positive triad. After adjusting for gender, age, and BMI, the severity of *H. pylori* infection were significantly associated with the higher risk observed for gastrointestinal symptoms (OR = 2.25, 95% CI: 1.91–2.69), sleep disturbances (OR = 2.86, 95% CI: 2.49–3.31), affective disorders (OR = 3.07, 95% CI: 2.64–3.60), and positive triad (OR = 3.19, 95% CI: 2.73–3.76). The exploratory subgroup analysis results are depicted in **Figure 4**. The effect of *H. pylori* infection on risk of positive triad was significant among different gender, age group, and BMI group.

**Table 2:** The association between *H. pylori* infection and different symptoms.

Characteristic	Unadjusted			Adjusted		
	OR*^1^*	95% CI*^1^*	*p*-value	OR*^1^*	95% CI*^1^*	*p*-value
Gastrointestinal Symptoms	2.24	1.90, 2.66	<0.001	2.25	1.91, 2.69	<0.001
Sleep Disturbance	2.90	2.53, 3.35	<0.001	2.86	2.49, 3.31	<0.001
Affective Disorders	3.05	2.63, 3.56	<0.001	3.07	2.64, 3.60	<0.001
Positive Triad	3.18	2.73, 3.73	<0.001	3.19	2.73, 3.76	<0.001

*^1^*OR, Odds Ratio; CI, Confidence Interval. Multivariate logistic regression analyses were performed, with the models adjusted for gender, age, and BMI.

## Discussion

The present study provides compelling evidence that *H. pylori* infection is significantly associated with a triadic symptom cluster comprising gastrointestinal discomfort, sleep disturbances, and affective disorders. Notably, among all participants, 60.7% exhibited this “Positive Triad” phenotype. Furthermore, higher levels of ^13^C urea breath test (^13^C-UBT) values—indicative of bacterial load—were significantly correlated with increased risk of symptom clustering, suggesting that the severity of infection may proportionally influence systemic symptom burden. These findings align with current perspectives in psychoneurogastroenterology, which emphasize the bidirectional interactions among the gut, brain, and circadian systems through shared neuroimmune and endocrine pathways (8, 21).

Beyond *H. pylori*, existing literature has consistently documented the frequent co-occurrence of gastrointestinal, sleep, and affective symptoms in various clinical populations. For instance, up to 66% of individuals with depression also experience insomnia, with sleep disturbances often preceding or exacerbating mood disorders (21, 22). In patients with irritable bowel syndrome (IBS), a prototypical functional gastrointestinal disorder, the prevalence of sleep disturbances has been estimated at 37.6%, while anxiety and depression are observed in approximately 39.1% and 28.8% of cases, respectively (23). National data from the United States further revealed that 73% of individuals with IBS reported sleep-related complaints, 37% had anxiety disorders, and 27% experienced affective disorders—substantially higher rates than in the general population (24). Similarly, in functional dyspepsia (FD), psychiatric and sleep comorbidities are widely reported. Studies indicate that more than half of FD patients present with depressive symptoms, and 70% with anxiety, while over 60% meet diagnostic criteria for insomnia or report poor sleep quality (25, 26). A Taiwanese population-based cohort further demonstrated that individuals with sleep disturbances had a four-fold increased risk of developing FD, and when combined with depression, the adjusted hazard ratio rose to 5.38—highlighting the compounding effects of emotional and sleep dysfunction on gastrointestinal outcomes (27).

These converging data support the concept of a trans-symptom cluster that is both clinically observable and biologically plausible. The GSRS (15 items; five domains; 1-week recall) demonstrates strong construct validity and internal consistency in multinational and Chinese samples; it quantifies symptom burden rather than offering diagnoses. The AIS (ICD-10 anchored) shows adequate-to-good internal consistency in Chinese cohorts and captures nocturnal and daytime impact, though it does not index objective sleep. The PHQ-4 (PHQ-2 + GAD-2) retains a stable two-factor structure with acceptable reliability in Chinese validations. It is worth mentioning that it prioritizes feasibility as an brief screener and across instruments, self-report and recall windows may introduce nondifferential misclassification. By integrating validated psychometric instruments with latent class analysis, we demonstrate that these symptoms frequently co-occur in a syndromic pattern, with infection severity correlating strongly with symptom burden. This finding aligns with emerging paradigms in psychoneurogastroenterology, which emphasize the interconnectedness of gut dysfunction, neuropsychiatric symptoms, and circadian dysregulation through shared biological pathways (28, 29). Our findings offer a critical step toward recognizing this interconnected phenotype and its relevance to *H. pylori*-related morbidity.

The observed triad resonates with recent advances in understanding *H. pylori*’s systemic effects. Chronic infection induces persistent gastric inflammation, characterized by elevated IL-6, TNF-α, and NF-κB activation (30), which may permeate the gut-brain axis via vagal afferents, circulating cytokines, and microbial metabolite signaling (8, 31). Notably, IL-6 and TNF-α are potent modulators of both serotonin metabolism and hypothalamic-pituitary-adrenal (HPA) axis activity, providing a plausible link to anxiety/depression scores in our cohort (32). Furthermore, *H. pylori*-induced dysregulation of gastric melatonin secretion—a key regulator of circadian rhythms—may directly impair sleep architecture, as evidenced by recent proteomic studies demonstrating suppressed MT1 receptor expression in infected gastric mucosa (33).

Our findings complement work on microbial regulation of the gut-brain axis. Some research showed *H. pylori* produces γ-glutamyl transpeptidase (GGT), which disrupts glutamatergic neurotransmission and blood-brain barrier integrity in animal models (34). This could explain the dose-dependent relationship between infection severity and PHQ-4 scores observed here. Additionally, the microbiome’s role in tryptophan metabolism—a precursor for both serotonin and melatonin—offers a unifying mechanism for the co-occurrence of mood and sleep disturbances (35).

The identification of this symptom triad challenges conventional diagnostic silos. While current guidelines focus on *H. pylori*’s role in peptic ulcers and gastric cancer (36), our data suggest that subclinical neuropsychiatric manifestations may precede overt gastrointestinal pathology. This aligns with recent proposals to classify *H. pylori* as a “systems pathogen” capable of inducing low-grade systemic inflammation with multi-organ consequences (37). Clinically, the strong association between ^13^C-UBT values and the “Positive Triad” (OR = 3.19) implies that breath test results could serve as biomarkers for predicting extra-gastric morbidity, particularly in populations with unexplained somatic symptom clusters.

Notably, our subgroup analyses revealed consistent associations across gender, age, and BMI strata—a finding with important public health implications. The heightened vulnerability in females parallels recent epigenomic studies showing estrogen-mediated amplification of *H. pylori*-induced IL-1β production (38), while the age-independent effects contradict earlier assumptions that infection sequelae diminish with immune senescence. These discrepancies highlight the need for lifespan-specific management strategies.

The symptom triad’s coherence with TCM’s holistic framework represents a novel conceptual advance. In TCM theory, “Liver-Qi stagnation affecting the Spleen-Stomach axis” manifests as digestive complaints, insomnia, and emotional instability—a pattern remarkably congruent with our findings. Modern mechanistic studies now validate these ancient observations: vagally-mediated gut-liver-brain communication pathways show increased activation in *H. pylori* infection (39), while microbiota-derived indole metabolites modulate hepatic cytochrome P450 enzymes involved in psychotropic drug metabolism (40). This convergence of Eastern and Western medical paradigms underscores the value of integrative models for understanding infection-related systemic dysfunction.

## Limitations

Nevertheless, several limitations should be acknowledged. Because recruitment occurred in a tertiary *H. pylori* detection clinic, selection bias is possible and may limit generalizability to community samples. This study have therefore framed all results as associations and recommend multi-center, population-based replication. The proportions of participants across *H. pylori* infection severity groups were not perfectly balanced, with a predominance of individuals classified as having severe H. pylori infection. This disproportion reflects the real-world clinical profile of patients attending tertiary diagnostic centers in high-prevalence regions of China (36, 41–43). While the overall statistical power was sufficient to detect meaningful differences, the relatively small size of the H. pylori-negative group may introduce bias and limit generalizability. In response to this concern, we conducted *post hoc* power analyses and confirmed adequate power based on the observed effect sizes. Still, future studies should consider more balanced recruitment strategies, and further validate our findings through multi-center studies with larger and more balanced cohorts to address this limitation.

While the current findings demonstrate a robust association between *H. pylori* infection severity and the presence of the gastrointestinal–sleep–affective symptom triad, the cross-sectional design precludes inference of temporal or causal relationships. Longitudinal studies are needed to investigate whether eradication of *H. pylori* leads to remission of these co-occurring symptoms, and whether the trajectory of triad resolution differs by baseline infection severity or symptom phenotype. Prospective cohorts and pre/post-eradication comparisons—ideally incorporating objective sleep measures and documented eradication history—are warranted to test mechanisms. Future research should explore the biological mechanisms underlying this triadic clustering. Future longitudinal studies should employ metagenomic sequencing to track *H. pylori* strain-specific effects and quantify microbial translocation markers to clarify gut-brain signaling mechanisms.

## Conclusion

This study provides preliminary evidence that *H. pylori* infection is associated with a triadic symptom cluster comprising gastrointestinal discomfort, sleep disturbances, and affective disorders. By integrating latent class modeling with systems biology perspectives, this study reveals that individuals with higher levels of *H. pylori* colonization face a markedly increased likelihood of experiencing co-occurring dyspeptic complaints, insomnia, and mood disturbances. Our study advances beyond symptom-centric approaches to propose a multi-domain symptom pathophysiological framework. Furthermore, this study highlights the importance of examining the triad of gastrointestinal symptoms, sleep disturbances, and affective disorders as an interconnected phenomenon. While causal relationships cannot be inferred from this cross-sectional design, the results underscore the need for greater clinical attention to the neuropsychiatric and sleep-related manifestations in H. pylori-positive individuals. Future longitudinal and mechanistic studies are warranted to confirm causality, clarify microbial-host signaling pathways, and explore the potential of integrated therapeutic strategies—such as microbiota modulation or circadian-targeted interventions which are ongoing in our follow-up studies—for symptom relief.

## Data Availability

The raw data supporting the conclusions of this article will be made available by the authors, without undue reservation.
