# Supplementary material for: Association of co-occurrence of gastrointestinal, sleep, and affective symptoms with Helicobacter pylori infection: a monocentric cross-sectional study in China
Source: Front Endocrinol (Lausanne). 2025 Nov 7;16:1675866. doi: 10.3389/fendo.2025.1675866 (PMC12634323; doi:10.3389/fendo.2025.1675866)
Supplement: Supplementary file 1 [file Table1.docx]

# Table 1. Gastrointestinal Symptom Rating Scale (GSRS)

Total Score: __________ Date: ______ Year ______ Month ______ Day Filled by: __________________

Instructions: This is a patient self-assessment questionnaire. The 15 items reflect five gastrointestinal symptom clusters: Reflux, Abdominal Pain, Indigestion, Diarrhea, and Constipation. Please recall your symptoms and sensations during the past 7 days, and tick “√” the option that best describes their severity.

In the past week, have you experienced the following symptoms or sensations?

| No. | Symptom Description | 1 No discomfort | 2 Slight | 3 Mild | 4 Moderate | 5 Rather severe | 6 Severe | 7 Very severe |
| --- | --- | --- | --- | --- | --- | --- | --- | --- |
| 1 | Pain or discomfort in the upper abdomen or chest | ☐ | ☐ | ☐ | ☐ | ☐ | ☐ | ☐ |
| 2 | Heartburn | ☐ | ☐ | ☐ | ☐ | ☐ | ☐ | ☐ |
| 3 | Acid regurgitation | ☐ | ☐ | ☐ | ☐ | ☐ | ☐ | ☐ |
| 4 | Hunger pain | ☐ | ☐ | ☐ | ☐ | ☐ | ☐ | ☐ |
| 5 | Nausea or vomiting | ☐ | ☐ | ☐ | ☐ | ☐ | ☐ | ☐ |
| 6 | Bowel sounds (borborygmi) | ☐ | ☐ | ☐ | ☐ | ☐ | ☐ | ☐ |
| 7 | Abdominal bloating | ☐ | ☐ | ☐ | ☐ | ☐ | ☐ | ☐ |
| 8 | Belching or hiccups | ☐ | ☐ | ☐ | ☐ | ☐ | ☐ | ☐ |
| 9 | Passing gas (flatulence) | ☐ | ☐ | ☐ | ☐ | ☐ | ☐ | ☐ |
| 10 | Constipation | ☐ | ☐ | ☐ | ☐ | ☐ | ☐ | ☐ |
| 11 | Diarrhea | ☐ | ☐ | ☐ | ☐ | ☐ | ☐ | ☐ |
| 12 | Loose or unformed stools | ☐ | ☐ | ☐ | ☐ | ☐ | ☐ | ☐ |
| 13 | Hard or pellet-like stools | ☐ | ☐ | ☐ | ☐ | ☐ | ☐ | ☐ |
| 14 | Urgency to defecate | ☐ | ☐ | ☐ | ☐ | ☐ | ☐ | ☐ |
| 15 | Sensation of incomplete evacuation | ☐ | ☐ | ☐ | ☐ | ☐ | ☐ | ☐ |
