# Supplementary material for: Association of co-occurrence of gastrointestinal, sleep, and affective symptoms with Helicobacter pylori infection: a monocentric cross-sectional study in China
Source: Front Endocrinol (Lausanne). 2025 Nov 7;16:1675866. doi: 10.3389/fendo.2025.1675866 (PMC12634323; doi:10.3389/fendo.2025.1675866)
Supplement: Supplementary file 2 [file Table2.docx]

# Table 2 Athens Insomnia Scale (AIS)

Please answer the following items based on your sleep over the past month.
Use a scale from 0 (no problem at all) to 3 (very serious problem).

| No. | Item | 0 No problem | 1 Mild problem | 2 Considerable problem | 3 Very serious problem |
| --- | --- | --- | --- | --- | --- |
| 1 | Difficulty with sleep induction (falling asleep) | ☐ | ☐ | ☐ | ☐ |
| 2 | Awakenings during the night | ☐ | ☐ | ☐ | ☐ |
| 3 | Early morning awakening | ☐ | ☐ | ☐ | ☐ |
| 4 | Total sleep duration | ☐ | ☐ | ☐ | ☐ |
| 5 | Overall quality of sleep | ☐ | ☐ | ☐ | ☐ |
| 6 | Sense of well-being during the day | ☐ | ☐ | ☐ | ☐ |
| 7 | Functioning (physical/mental) during the day | ☐ | ☐ | ☐ | ☐ |
| 8 | Sleepiness during the day | ☐ | ☐ | ☐ | ☐ |

Scoring:

Each item is scored from 0 to 3. Total score ranges from 0 to 24.
- A total score of 6 or more is suggestive of insomnia.
- Items 1–5 assess night-time sleep, and items 6–8 assess daytime dysfunction.
