# Supplementary material for: Association of co-occurrence of gastrointestinal, sleep, and affective symptoms with Helicobacter pylori infection: a monocentric cross-sectional study in China
Source: Front Endocrinol (Lausanne). 2025 Nov 7;16:1675866. doi: 10.3389/fendo.2025.1675866 (PMC12634323; doi:10.3389/fendo.2025.1675866)
Supplement: Supplementary file 3 [file Table3.docx]

# Table 3 Patient Health Questionnaire-4 (PHQ-4)

Over the last 2 weeks, how often have you been bothered by the following problems?
Please tick the box that best describes your experience.

| No. | Item | Not at all (0) | Several days (1) | More than half the days (2) | Nearly every day(3) |
| --- | --- | --- | --- | --- | --- |
| 1 | Feeling nervous, anxious or on edge | ☐ | ☐ | ☐ | ☐ |
| 2 | Not being able to stop or control worrying | ☐ | ☐ | ☐ | ☐ |
| 3 | Little interest or pleasure in doing things | ☐ | ☐ | ☐ | ☐ |
| 4 | Feeling down, depressed or hopeless | ☐ | ☐ | ☐ | ☐ |

Scoring:

Each item is scored from 0 to 3, total score ranges from 0 to 12.
- Items 1-2 form the GAD-2 (Generalized Anxiety Disorder) scale.
- Items 3-4 form the PHQ-2 (Depression) scale.
